# Fluorescent Protein Voltage Probes Derived from ArcLight that Respond to Membrane Voltage Changes with Fast Kinetics

**DOI:** 10.1371/journal.pone.0081295

**Published:** 2013-11-27

**Authors:** Zhou Han, Lei Jin, Jelena Platisa, Lawrence B. Cohen, Bradley J. Baker, Vincent A. Pieribone

**Affiliations:** 1 Cellular and Molecular Physiology, Yale University School of Medicine, New Haven, Connecticut, United States of America; 2 The John B. Pierce Laboratory, Inc., New Haven, Connecticut, United States of America; 3 Faculty of Physical Chemistry, University of Belgrade, Belgrade, Serbia; 4 Center for Functional Connectomics, Korea Institute of Science and Technology, Seoul, Republic of Korea; 5 Neurobiology, Yale University School of Medicine, New Haven, Connecticut, United States of America; Indiana University School of Medicine, United States of America

## Abstract

We previously reported the discovery of a fluorescent protein voltage probe, ArcLight, and its derivatives that exhibit large changes in fluorescence intensity in response to changes of plasma membrane voltage. ArcLight allows the reliable detection of single action potentials and sub-threshold activities in individual neurons and dendrites. The response kinetics of ArcLight (τ1-on ~10 ms, τ2-on ~ 50 ms) are comparable with most published genetically-encoded voltage probes. However, probes using voltage-sensing domains other than that from the *Ciona intestinalis* voltage sensitive phosphatase exhibit faster kinetics. Here we report new versions of ArcLight, in which the *Ciona* voltage-sensing domain was replaced with those from chicken, zebrafish, frog, mouse or human. We found that the chicken and zebrafish-based ArcLight exhibit faster kinetics, with a time constant (τ) less than 6ms for a 100 mV depolarization. Although the response amplitude of these two probes (8-9%) is not as large as the *Ciona*-based ArcLight (~35%), they are better at reporting action potentials from cultured neurons at higher frequency. In contrast, probes based on frog, mouse and human voltage sensing domains were either slower than the *Ciona*-based ArcLight or had very small signals.

## Introduction

Genetically-encoded fluorescence protein (FP) voltage probes convert voltage changes across biological membranes into optical signals [1]. In principle, these probes allow for electrode-free electrophysiology of genetically-targeted cell types. Several design concepts have been used in engineering FP-based voltage probes. These include conjugating the voltage-sensing domains of membrane proteins with single FPs [1-3], Förster resonance energy transfer (FRET) pairs [4-7], split FPs [8] or circularly permuted FPs [9,10]. A different concept for a genetically-encoded voltage sensitive probe is based on the fluorescence of microbial rhodopsins [11,12]. However, the above probes have not found practical use because of one or more limitations, including small response amplitude, slow dynamics, low brightness, unwanted physiological activity and/or poor membrane localization e.g. [13]. 

The recently published FP voltage probe, ArcLight, based on fusion of the *Ciona intestinalis* voltage-sensing domain with super ecliptic pHluorin A227D, exhibited significantly improved response amplitudes (-30 to -40% ΔF/F in response to a 100mV depolarization) in mammalian cells [14]. Linker length optimized derivatives of this probe (ArcLight-Q239 and A242) readily resolve action potentials and sub-threshold electric events in cultured hippocampal neurons in single trials. ArcLight has also been used to record sensory responses *in vivo* in the mouse olfactory bulb (Storace et al., 2013 Biophysical Society abstract), mouse barrel cortex (Platisa and Pieribone, unpublished data), individual *Drosophila* neurons [15] and *C. elegans* (Wooltorton et al., Biophysical Society 2013 abstract). 

In some experimental situations, a voltage sensing probe with fast response kinetics would be useful. Unfortunately, the response kinetics of the *Ciona*-based ArcLight is relatively slow compared with small molecule voltage sensitive dyes [16] or some microbial rhodopsin-based probes [11]. Baker et al. (2012) demonstrated that Zahra, a FP voltage probe based on the zebrafish (*Danio rerio*) voltage-sensing domain, has fast kinetics, although its signal to noise characteristics are likely too small for use *in vivo* [17]. Two other voltage probes based on circularly permuted FPs or a chimera between the *Ciona* voltage-sensing domain and Kv3.1 also exhibit fast kinetics [9,18], but their application is limited by relatively small response amplitudes and/or poor membrane localization.

In this study, we sought to combine the large signal size afforded by super ecliptic pHluorin A227D with the speed improvements seen with voltage sensing domains from other phosphatase orthologs. We replaced the *Ciona* voltage-sensing domain in ArcLight with voltage-sensing domain orthologs from five species: chicken, frog, zebrafish, mouse and human. We found that probes utilizing the chicken or zebrafish voltage-sensing domain exhibited fast responses, and a larger response amplitude than Zahra. We demonstrated that chicken ArcLight can resolve individual action potentials in cultured neurons as well as trains of high frequency voltage steps in the HEK293 cells. The increase in response amplitude afforded by the ArcLight’s super ecliptic pHluorin A227D can be transferred to probes utilizing other voltage sensors and is capable of capturing rapid movements of the voltage sensing domain. These new probes should be useful for detecting fast and high frequency voltage changes in excitable cells.

## Results

### Comparison of ArcLight derived probes based on voltage-sensing domain orthologs from different species

We replaced the *Ciona* voltage-sensing domain used in the previously published ArcLight probe with voltage-sensing domain orthologs from five species: chicken (*Gallus gallus*, GenBank accession number XP_417079.2), frog (*Xenopus laevis*, Xl-VSP1, GenBank accession number NP_001090072.1), zebrafish (*Danio rerio*, GenBank accession number NP_001020629.1), mouse (*Mus musculus*, GenBank accession number NP_954866.2) and human (*Homo sapiens*, Hs-TPTE, GenBank accession number NP_954863.2). The fusion sites between the voltage-sensing domain and super ecliptic pHluorin A227D in these probes were: Q174 for the frog, Q175 for the chicken and zebrafish, Q331 for mouse and Q193 for human. The fusion sites were chosen to correspond to the fusion site of the *Ciona*-based ArcLight-Q239 probe (see Figure S1 for an alignment of voltage sensitive domains from the different species). An arginine to glutamine point mutation was introduced in the S4 domains (Figure S1), corresponding to the R217Q mutation found in the *Ciona* voltage-sensing domain in the original ArcLight probe. This mutation causes a shift of the F-V curve of the voltage probes leftward to a more physiological range [14,19]. 

**Figure 1:**
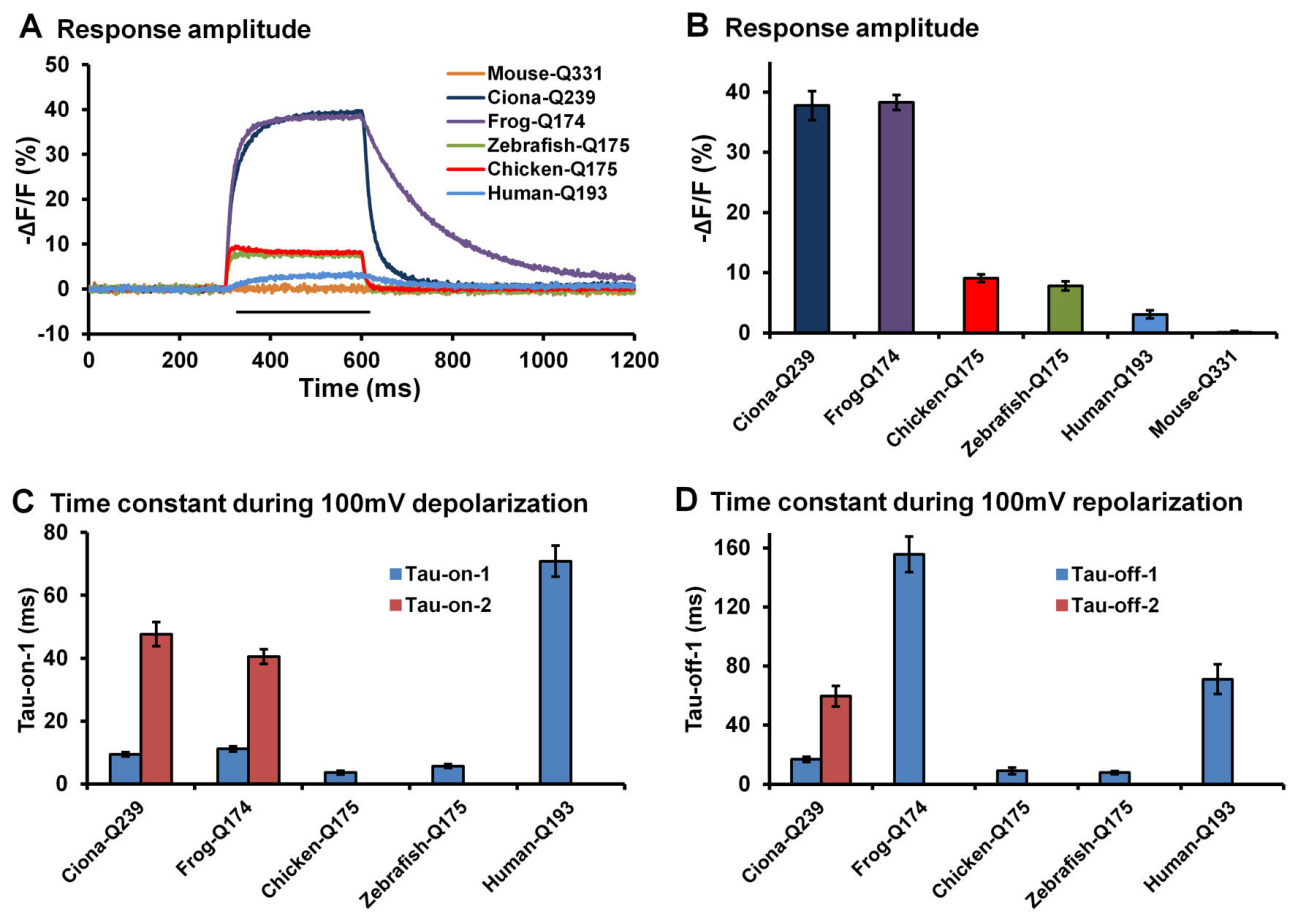
Comparison of response amplitude and kinetics of *Ciona* based ArcLight and ArcLight based on voltage-sensing domain orthologs from other species. A). Averaged fluorescence traces of *Ciona*-based ArcLight-Q239 and derivatives based on chicken, zebrafish, frog, mouse and human voltage sensitive phosphatase orthologs in response to 300 ms depolarizations of 100 mV (indicated by the black line) from a holding potential of -70 mV. The traces were recorded in HEK293 cells. *Ciona*: n=6; frog: n=6; chicken: n=6; zebrafish: n=8; mouse: n=4; human: n=6. The fusion sites for the voltage sensing domains were: Q239 for the *Ciona* voltage sensitive domain, Q175 for the chicken and zebrafish voltage-sensing domain, Q174 for the frog voltage sensitive domain, Q331 for the mouse voltage sensitive domain and Q193 for the human voltage sensitive domain. B). Averaged –ΔF/F of the traces in panel A. C). τ-on of the five probes in response to a 100 mV depolarization. D). τ-off of the five probes during repolarization. *Ciona* and frog based ArcLight were best fit with double exponentials for the “on” response. Chicken, zebrafish and human based ArcLight were well fit with a single exponential.

Tested in HEK293 cells with 100mV depolarization steps, the chicken ArcLight-Q175 exhibited a fluorescence intensity change of -9 ± 0.7% (Figure 1A and 1B). The kinetics of the fluorescence change of this probe, however, were both simpler and much faster (τ-on ~ 4ms, τ-off ~ 9ms; Figure 1) compared to all other *Ciona*-based FP voltage sensors, including the ArcLight-Q239 probe (τ1-on ~ 10ms, τ1-off ~ 20ms; Figure 1C and 1D) [14]. The zebrafish ArcLight-Q175 produced a fluorescence response (-8 ± 0.7%) that has similar kinetics (τ-on ~ 6 ms, τ-off ~ 8 ms; Figure 1) as chicken ArcLight. In contrast, the *X. laevis*-based probe generated a large but slow response (-38 ± 1%; τ-on ~ 11ms, τ-off ~ 155ms; Figure 1), even slower than that seen with the *Ciona*-based ArcLight. The human-based probe responded to a 100mV depolarization with a small and slow response (-2 ± 1%; τ-on and τ-off ~ 70ms; Figure 1). The mouse-based probe has no detectable responses to a 100mV depolarization (Figure 1A). Unlike *Ciona*-based probes the “on” and “off” kinetics of chicken ArcLight-Q175 were well fit with single exponential curves; there was no improvement in fit with a double exponential.

**Figure 2:**
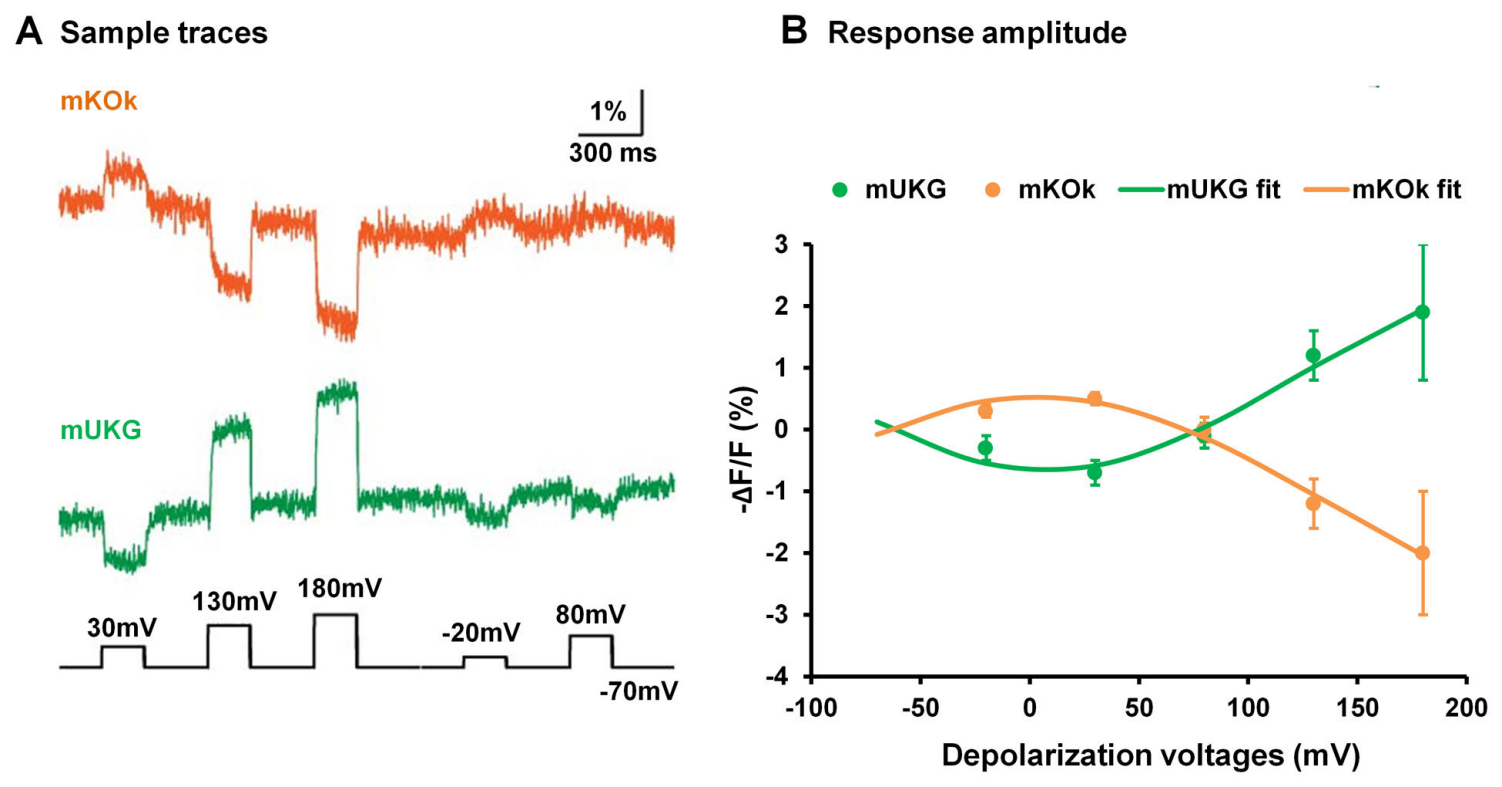
Fluorescence changes of a probe based on the chicken voltage-sensing domain and the Mermaid FRET pair (mUKG-mKOk). A). Averaged optical traces of ten trials of this probe from a HEK 293 cell in response to voltage clamp depolarization steps. The fusion site was at chicken voltage sensitive domain S188, corresponding to *Ciona* ArcLight-S249 (Figure S1). The data is corrected by exponential subtraction and Gaussian low-pass filter of 210 Hz. B). The ΔF/F *vs* V plot of this probe. Averaged data from multiple cells were used in the analysis. -20mV: n=2; 30mV: n=4; 80mV: n=2; 130mV: n=3; 180mV: n=2. The data did not fit well with the Boltzmann equation, but were best fit with the area version of the Gaussian function.

When the chicken voltage sensing domain is fused with the FRET pair, mUKG and mKOk, from the Mermaid voltage probe [4], the resulting probe exhibited similar kinetics to its super ecliptic pHluorin A227D counterpart but with a much reduced response amplitude (Figure 2). The fluorescence responses of the “chicken mUKG/mKOk” probe in response to different depolarizations were not always in the same direction (Figure 2), a response pattern not seen previously. 

### Effect of Inker Length Modification on Chicken ArcLight

**Figure 3:**
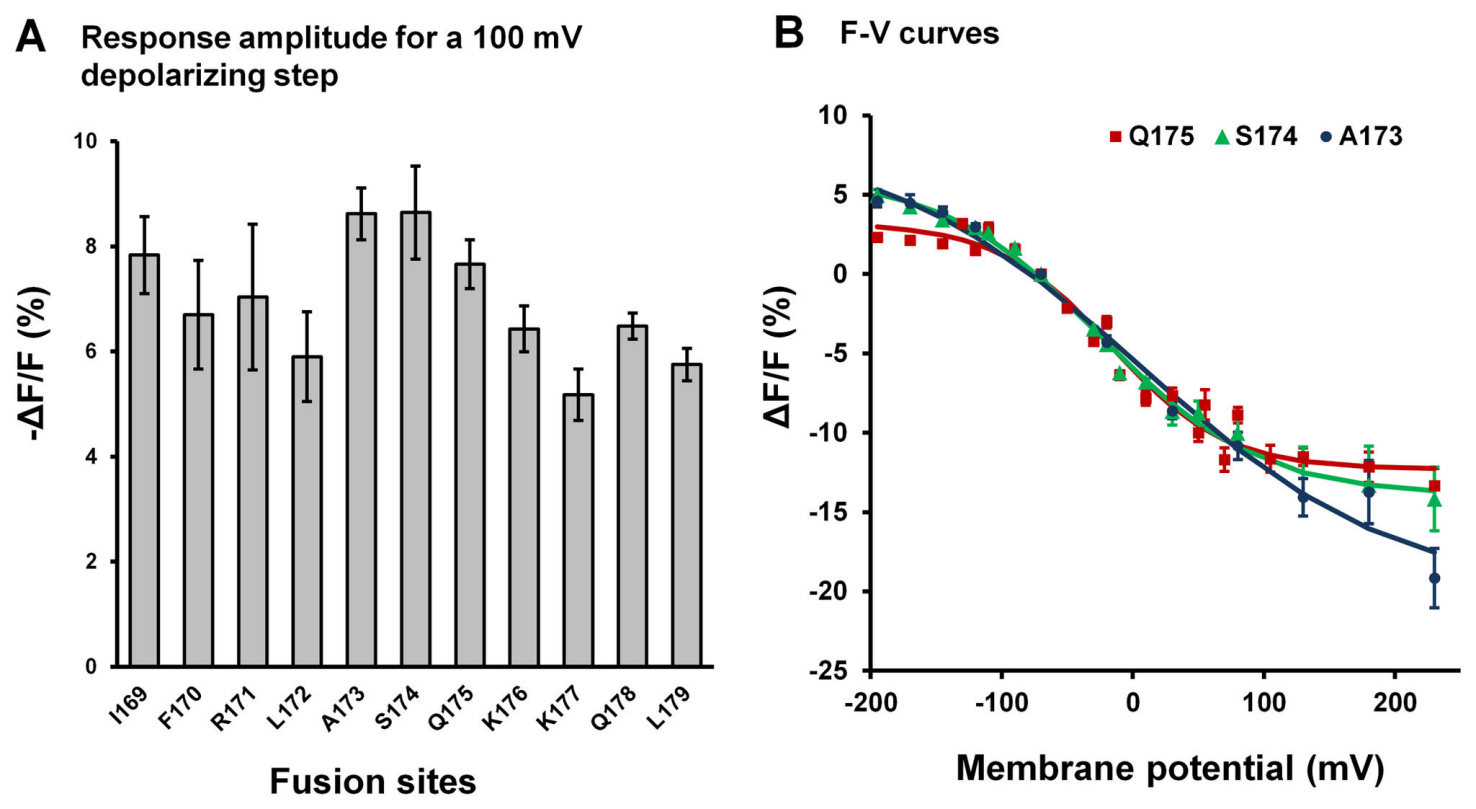
Effect of linker length on the chicken ArcLight signal. A). Response amplitude of probes resulting from fusion of super ecliptic pHluorin A227D with the chicken voltage sensitive domain at different sites. The membrane potential was held at -70mV and fluorescence changes were recorded in response to a 300 ms depolarization step of 100 mV. I169: n=4; F170: n=4; R171: n=3; L172: n=4; A173: n=13; S174: n=7; Q175: n=20; K176: n=10; K177: n=9; Q178: n=10; L179: n=10. B). Superimposed F-V curves of linker modified chicken ArcLight at position A173, S174 and Q175.

Fusing super ecliptic pHluorin A227D to the *Ciona* voltage-sensing domain at different positions along the linker alters the signal size of ArcLight, with the maximum signal achieved when the FP is fused to the *Ciona* voltage-sensing domain at positions from Q239 to A242 [14]. We sought to determine if the modulatory effect of the linker length can also be seen in ArcLight derivatives based on voltage sensitive phosphatase orthologs from other species. Eleven probes were engineered with super ecliptic pHluorin A227D fused to the S4 domain of the chicken voltage-sensing domain at different sites, i.e. after amino acids I169, F170, R171, L172, A173, S174, Q175, K176, K177, Q178 and L179 (Figure S1). The size of the voltage dependent fluorescence changes in these probes in response to 100mV depolarization steps is shown in Figure 3A. The largest signals, -8.6 ± 1%, were obtained by fusing the FP at S174 of the chicken voltage-sensing domain (Figure 3A). Fusions at positions I169 and F170 resulted in probes with slowest voltage sensitivity of the tested probes (data not shown). The V_1/2_ of the F-V curves of these linker modified derivatives of chicken ArcLight was located in the physiological range. We present the F-V curves of Chicken ArcLight-A173, S174 and Q175 as examples (Figure 3B).

### Evoked action potentials recorded with chicken ArcLight

**Figure 4:**
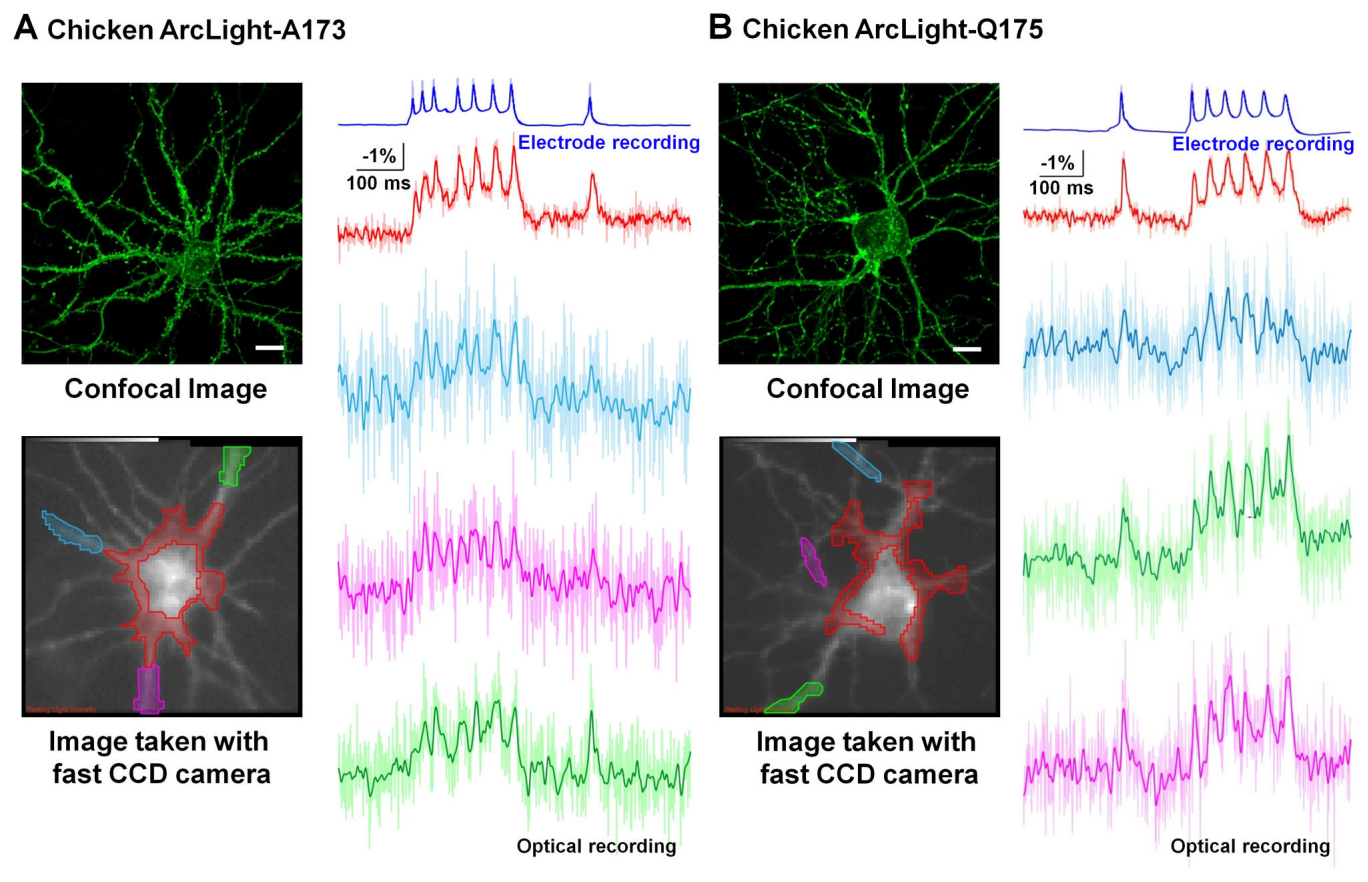
Single action potentials were resolved with chicken ArcLight in cultured cortical neurons. A). Top left panel: Confocal image of chicken ArcLight-A173 expressed in an acutely cultured mouse cortical neuron. Scale bar = 20 μm. Bottom left panel: An 80x80 fast CCD camera image of a neuron, from which the action potentials in the right panel were recorded. The ROIs were shaded with the matching colors to the traces. Right panel: Single trial recordings of evoked action potentials using chicken ArcLight-A173. Both the optical recording around the soma and electrode recordings were low-pass filtered with a 95 Hz Gaussian filter. The processes’ optical signals were low-pass filtered with a 50 Hz Gaussian filter. The un-filtered traces are shown as the light-colored lines. Bleaching was corrected with exponential subtraction. B). Top left panel: Confocal image of chicken ArcLight-Q175 expressed in an acutely cultured mouse cortical neuron. Scale bar = 20 μm. Bottom left panel: An 80x80 fast CCD camera image of a neuron, from which the action potentials in the right panel were recorded. The ROIs were shaded with the matching colors to the traces. Right panel: Single trial recordings of evoked action potentials using chicken ArcLight-Q175. Both the optical recording around the soma and electrode recordings were low-pass filtered with a 108 Hz Gaussian filter. The processes’ optical signals were low-pass filtered with a 50 Hz Gaussian filter.

**Figure 5:**
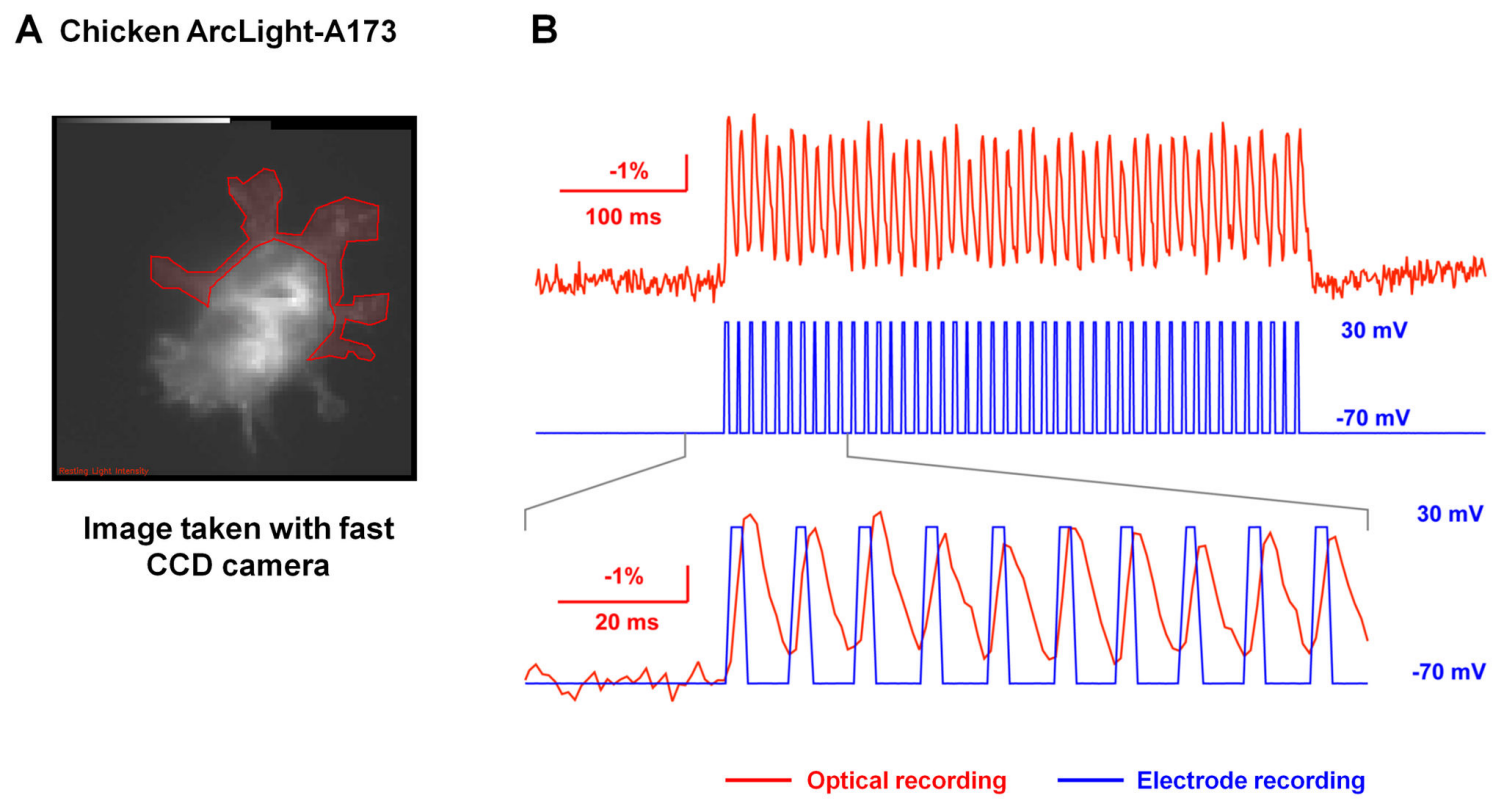
Chicken Arclight-A173 resolves 100 Hz, 2 ms depolarization pulses. A). Two HEK 293 cells transfected with chicken ArcLight-A173 imaged with the fast CCD camera. The patched cell is in the top-right of this image. The ROI is shown in red. B). The red trace is –ΔF/F of the optical recording. The blue trace is the command voltage pulses. Overlapping the traces shows the temporal relationship between the optical recording and the command voltage pulses. It is most likely that the membrane was not fully charged during the pulse. We estimated that the actual stepped potential to be on the order of 70mV [17].

Expression of chicken ArcLight-A173 and Q175 in cultured mouse cortical neurons produced brightly fluorescent cells with membrane targeted expression in the soma and dendrites (Figure 4A and B). They appear largely membrane localized, similar to the *Ciona*-based ArcLight. In spite of the relatively small response amplitude of the chicken ArcLight in HEK 293 cells (~9% ΔF/F in response to a 100mV depolarization), we could detect evoked action potentials in single trials in neurons expressing this probe (Figure 4). The mean soma response was -3.2 ± 2.2% change in the fluorescence intensity (n=20 cells). We also tested whether chicken ArcLight is able to resolve voltage signals at a high frequency. We applied 100Hz depolarizations of 2 ms duration to HEK293 cells transfected with chicken ArcLight-A173 and these signals were resolved in single trials (Figure 5). 

We also compared chicken ArcLight with Arch and Arch D95N, both are microbial rhodopsin-based probes with fast kinetics [11]. We measured that the resting fluorescence of these two probes was about 100 times smaller than that for chicken ArcLight. After averaging 10 trials, we were not able to detect a fluorescence change of Arch or Arch D95N in response to 100 mV depolarization steps in HEK293 cells with the optical recording system we used (see Methods).

## Discussion

The action potential fractional changes, ΔF/F, with chicken ArcLight were the same as those found with the *Ciona*-based ArcLight [14], even though the changes in response to a 300 ms depolarizing step of 100 mV was ~4 times smaller for the chicken ArcLight (Figure 1A and B) . The faster response kinetics of the chicken sensor allows the signal to come closer to the steady state value during an action potential. The faster kinetics also results in a more rapid optical time course during an action potential (Figures 4 and 5).

We previously reported that voltage sensing domain orthologs from different species have faster response kinetics [17]. The current findings replicate and extend those findings and produced probes with larger signal to noise ratios. Fast kinetics are also seen in *Ciona*-based probes carrying different FP reporters (i.e. the Mermaid FRET pair; U. Sung, L. B. Cohen, Masoud Allaverdizadeh and B. Baker, unpublished observations). It is unclear why the voltage sensing domain of different species would result in reporters with different kinetics. It appears that the coupling between the voltage sensor and the fluorescent protein favors faster kinetics in some species. 

The chicken and zebrafish ArcLight have similar response kinetics as Zahra, but the response amplitude is larger (-9% for chicken and -8% for zebrafish ArcLight compared with -2% for Zahra). While the mechanism of the superior signal size of the ArcLight voltage probe is unknown, the current study demonstrates that the relatively large signal resulting from the use of super-ecliptic pHluorin A227D is transferrable to other voltage probes containing voltage sensing domain orthologs from different species. Probes containing super-ecliptic pHluorin A227D produce larger signals when compared to similar probes using other FPs (CFP/YFP in Baker, Jin et al. 2012 or mUKG and mKOk in Tsutsui, Karasawa et al. 2008). In addition it demonstrates that this enhanced modulatory behavior can respond rapidly. These finding indicate the portability of this fluorescent protein and its mechanism to other probe designs. 

All previously published *Ciona*-based voltage sensitive probes have a point mutation in the S4 transmembrane region that changes arginine to glutamine (R217Q) and causes a shift of the probe’s voltage dependent fluorescence change to a more physiological range [14,19]. The present probes contained the same point mutation in the corresponding location of S4 (R153Q for the chicken and zebrafish voltage sensitive domain, R152Q for the *X. laevis*, R309Q for the mouse and R171Q for the human) with a similar effect. Although we cannot be sure whether this effect is due to a change in sensing *per se* or in the coupling between the voltage-sensing domain and the fluorescent protein [20], a similar effect detected in probes using several orthologs suggest modification in the voltage sensitivity rather than an effect on the fluorescent proteins.

The frog ArcLight used for this study is based on Xl-VSP1, one of the two voltage sensitive phosphatase isoforms found in *X. laevis* [21]. This probe exhibits a fast increase during depolarization (τ-on-1 ~ 10 ms) but a very slow return to the baseline (τ-off ~ 150 ms) (Figure 1C-D). The response profile of frog ArcLight may result in a stair-like increase of signal to a train of action potentials. The final level reached during one behavioral event would then be an integral of the spike activity. Long-lasting signals have the additional advantage that a lower frequency cut-off low pass filter can be used which will improve the signal-to-noise ratio. 

The human ArcLight used for this study is based on Hs-TPTE, one of the two voltage sensitive phosphatase isoforms found in humans [22,23]. Hs-TPTE lacks the catalytic activity due to sequence changes in the enzyme active site [22]. One study showed that Hs-TPTE contains a functional voltage sensor, similar to that in non-mammalian orthologs [24]. Hs-TPTE also functions as a voltage-gated proton channel that is not observed in the zebrafish ortholog [24]. Since the “sensing current” is preserved in Hs-TPTE, it is hard to explain why a probe based on this protein lacks voltage sensitivity (Figure 1). Similar results were observed with a probe based on voltage-sensing domain from mouse (Figure 1). It is likely that the lack of sensitivity detected for probes based on the voltage sensing domain from human or mouse is due to their poor targeting to the plasma membrane, as was shown in Figure S2. It was reported that mammalian orthologs of these proteins appear to be restricted to the Golgi complex, both in spermatogenic cells and following expression in cell lines [22,25,26].

A single FP, super ecliptic pHluorin A227D, is used in ArcLight and in the newly developed chicken ArcLight. Although FRET based FP sensors enable ratiometric processing of signals and cancelation of bleaching or movement artifacts, there are also advantages for using a single FP as the reporter. One advantage is that it reduces the size of constructs needed for the viral vector delivery [27,28]. 

Arch (Addgene Plasmid 22217) and Arch D95N (Addgene Plasmid 34616) are microbial rhodopsin based fluorescence voltage probes and feature fast response to membrane voltage changes and slow bleaching rates [11]. However, the quantum yield of both Arch and Arch D95N is very small, 4x10^-4^ and 9x10^-4^, respectively [11]. As a result, the read noise was the dominant noise source in the Arch measurements. We were unable to detect a fluorescence change of either probe in HEK293 cells with our optical setup.

In the future, it may be necessary to exploit the voltage-sensing domain from more species in designing improved FP voltage sensors. It may also be useful to introduce mutations to the voltage sensitive domain, especially to the transmembrane domains in screening for probes with fast kinetics. 

## Methods

### Molecular biology

ArcLight derivatives based on the voltage sensing domains from voltage sensitive phosphatases from three species were generated by replacing the *Ciona* voltage-sensing domain (R217Q) in ArcLight with voltage sensitive domains from chicken, *X, laevis*, zebrafish, mouse or human. The coding sequence of the voltage sensitive domains were amplified with the polymerase chain reaction (PCR) using the pfu DNA polymerase (Agilent Technologies, Inc., CA), digested with restriction enzymes (HindIII and BamHI for chicken and *X. laevis* voltage sensitive domain, HindIII and BglII for zebrafish voltage sensitive domain) and inserted between the HindIII and BamHI sites of the ArcLight construct. An extra codon GAG was introduced immediately after the starting ATG to create an optimal Kozak consensus sequence. Primers used for amplification of chicken voltage-sensing domain were:

5: GCGAAGCTTCGCCACCATGGAGACGACTGTGAGGTATGAACAG-3 and 

5- CCGGGATCCCCAGAGACCATTCTTCTGGTTAC-3. Primers used for amplification of *X. laevis* voltage-sensing domain were: 5-GCCGCTAGCGCCACCATGGCCTCAAGTGTAACAGACGAGCAG-3 and 5-CCGGGATCCCCAGAGACCAGCCTCCTTGTTAC-3. Primers used for amplification of zebrafish voltage-sensing domain were: 5-GCCGCTAGCGCCACCATGGCCACGTCTGTGCATTTTAACCCTG-3 and 5-CCGAGATCTCCTGAAACCATTCTCCTGGTGAC-3. Primers used for amplification of mouse voltage-sensing domain were: 5-GCGAAGCTTCGCCACCATGTATGGAGAAAAGAAGAGCCATTTGTATCTC-3 and 5-CGCGGATCCCCTTGATGAGCCAGTTGCAGAATCC-3. Primers used for amplification of human voltage-sensing domain were: 5-GCGAAGCTTCGCCACCATGGAGAATGAAAGTCCTGATCC-3 and 5-CGCGGATCCCCATGAAACAGATGAAAAATTCTTAACAG-3. An arginine to glutamine mutation was introduced to the S4 of the voltage-sensing domains (R153Q for the chicken and zebrafish, R152Q for the *X. laevis*, R309Q for the mouse and R171Q for the human), corresponding to the location of R217Q in ArcLight. Point mutation and modification to the linker sequences between the chicken voltage-sensing domain and super ecliptic pHluorin were introduced by using the QuickChange II XL site-directed mutagenesis kit (Agilent Technologies, INC., CA). All DNA constructs were verified by sequencing using the dye-termination method (W. M. Keck Foundation, Biotechnology Resource Laboratory, Yale University, CT)

### Cell culture

HEK 293 cells (AATC, VA) were maintained in Dulbecco’s Modified Eagle Medium (High Glucose; DMEM; Invitrogen, NY) supplemented with 8% fetal bovine serum (FBS; Invitrogen, NY). Cortical neurons were isolated from E18 mouse embryos and maintained in Neurobasal medium with 0.5 mM Glutamax-I and 1 ml of B-27 supplement (Invitrogen, NY) per 50 ml of cultured medium. Cells were plated on coverslips coated with poly-D-lysine hydrobromide (MP Biomedicals, OH) and kept in an incubator at 37°C with 5% CO2. Transient transfection was accomplished by using half of the manufacturer’s recommended amount of DNA (2 μg per 35 mm dish or 0.4 μg per 12 mm coverslip in 24-well dish) and Lipofectamine2000 (1 μl per 12mm coverslip; Invitrogen, NY).

### Electrophysiology

Microelectrode recordings were performed in a perfused chamber with the bath temperature kept at 33°C by a temperature controller and bath solution containing: 150 mM NaCl, 4 mM KCl, 2 mM CaCl2, 1 mM MgCl2, 5 mM D-glucose, and 5 mM HEPES, pH7.4. We used a 3-5 MΩ glass patch pipettes (capillary tubing with 1.5/0.75 mm OD/ID-World Precision Instruments, FL) that were pulled on a P-97 Flaming/Brown type micropipette puller (Sutter Instrument Company, CA). The pipette solution contained 120 mM K-aspartate, 4 mM NaCl, 4 mM MgCl2, 1mM CaCl2, 10 mM EGTA, 3 mM Na2ATP and 5 mM HEPES, pH 7.2. Voltage-clamp recordings in the whole-cell configuration were performed using a Patch Clamp PC-505B amplifier (Warner Instruments, CT) with a holding potential of -70 mV. Cultured cortical neurons were recorded in current clamp mode. Action potentials were evoked by current injections. The pipette solution for neuron recordings contained: 120 mM K-gluconate, 3 mM

KCl, 7 mM NaCl, 4 mM Mg-ATP, 0.3 mM Na-GTP, 20 mM HEPES and 14 mM Trisphosphocreatin, pH adjusted with KOH to pH 7.3[29]. The sensor response time constants (τ) are the measured values. They are not corrected for the time required for the voltage change (1-2 ms) of our voltage clamp system [17].

### Wide field imaging

Whole-cell patch clamped cells were imaged with a Nikon Eclipse E6000FN upright microscope with a water immersion objective, Nikon Fluor 60×/1.00 N.A. A MLL-FN-473 nm 50 mW (Changchun New Industries Optoelectronics Tech. Co., Ltd., China) was used as the excitation light source. The laser light was transmitted into the microscope by a multi-mode fiber coupler (Siskiyou, OR), a quartz light guide and an Achromatic EPI-Fluorescence Condenser (Till Photonics, NY). The filter cube contains a dichroic mirror 505DCXR and an emission filter HQ510LP (Chroma, Bellows Falls, VT). The fluorescence image was demagnified by an Optem® zoom system, A45699 (QioptiqLINOS Inc, NY) and projected onto the 80×80 pixel chip of a NeuroCCD-SM camera controlled by NeuroPlex software (RedShirtImaging, GA). The images were recorded at a frame rate of 1000 fps. 

### Confocal imaging

Confocal images were obtained with a Zeiss 780 LSM (Carl Zeiss AG, Germany) confocal laser scanning microscope using a Plan-Apochromat 63x/1.40 Oil DIC M27 objective. A 488 nm wavelength Argon laser was used for chromophore excitation. The dichroic beam splitter was a MBS 488. The emission filter was 493 - 598nm. Zeiss Zen 2009 software was used for image acquisition and processing.

### Data processing

NeuroPlex software (RedShirtImaging, GA) was used to view the image sequences and output optical and electrophysiological recordings. The % ΔF/F was calculated by first subtracting the dark image from all frames, then the average of a region of interest in each frame (F) is subtracted from the average of the region taken from one hundred frames prior to the event of interest (F0) and this value is then divided by F0, i.e. % ΔF/F = ((F-F0) / F0)*100. The data were further processed and statistically analyzed with Origin8.1 (OriginLab, MA) and Excel (Microsoft, WA).

The probe dynamics are fitted with either a single exponential equation: 

$$y=y_{0}+a_{1}e^{-\left(x-x_{0}\right)/\tau_{1}}$$

or a double exponential equation:

$$y=y_{0}+a_{1}e^{-\left(x-x_{0}\right)/\tau_{1}}+a_{2}e^{-\left(x-x_{0}\right)/\tau_{2}}$$

The ΔF/F *vs* V plot was analyzed with the Boltzmann equation:

$$y=a_{2}+\frac{a_{2}-a_{1}}{1+e^{\left(x-x_{0.5}\right)/dx}}$$

where x_0.5_ is the membrane potential in mV at half maximal ΔF/F, and dx is the slope.

The normalized ΔF/F *vs* V plot was calculated from the Boltzmann fit: 

$$y=\frac{1}{1+e^{\left(x-x_{0.5}\right)/dx}}$$

The ΔF/F *vs* V plot of a probe based on the chicken voltage-sensing domain and the Mermaid FRET pair was calculated with the area version of the Gaussian function:

$$y=y_{0}+\frac{A}{w\sqrt{\frac{\pi }{2}}}e^{-2{\left(\frac{x-xc}{w}\right)}^{2}}$$

## Supporting Information

Figure S1 **Protein sequence alignment of N-terminal regions of the voltage sensitive phosphatase from six species.** Conserved residues are shaded. Transmembrane domains are indicated with orange and the S#. The arginine to glutamine point mutation that shifts the F-V curve of the probes to more physiological range is marked in red. The fusion site corresponding to that of ArcLight-Q239 is highlighted with yellow.
(TIF)

Figure S2 **Expression of human or mouse ArcLight in transfected HEK 293 cells.** Representative confocal images of human ArcLight-Q193 or mouse ArcLight Q331 expression in the HEK 293 cells. The probes carry either an arginine to glutamine mutation or not in the S4 domain.
(TIF)
