# Supplementary figures and images for: Fluorescent Protein Voltage Probes Derived from ArcLight that Respond to Membrane Voltage Changes with Fast Kinetics
Source: PLoS One. 2013 Nov 27;8(11):e81295. doi: 10.1371/journal.pone.0081295 (PMC3842285; doi:10.1371/journal.pone.0081295)

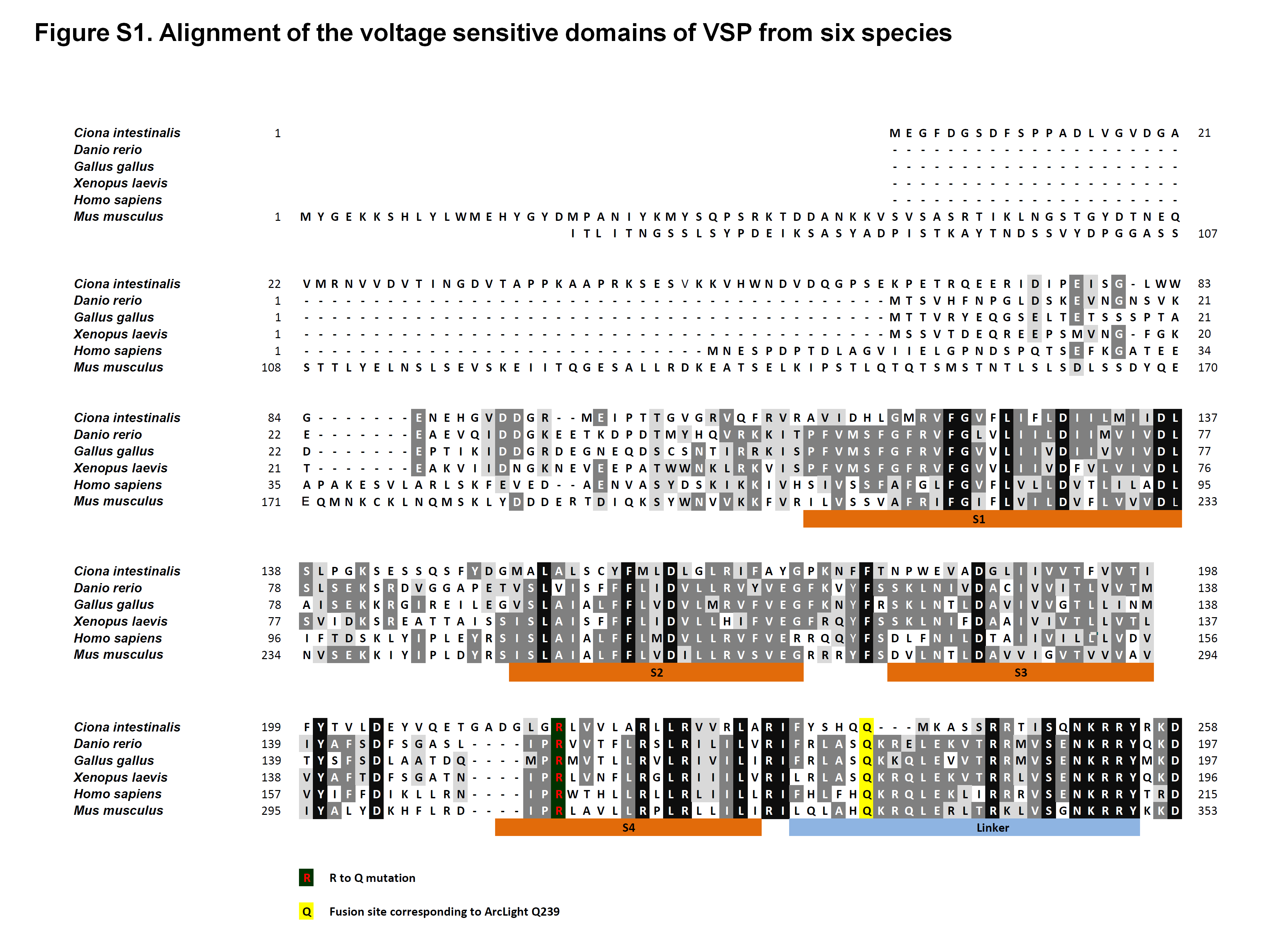

Supplement: Figure S1 — Protein sequence alignment of N-terminal regions of the voltage sensitive phosphatase from six species. Conserved residues are shaded. Transmembrane domains are indicated with orange and the S#. The arginine to glutamine point mutation that shifts the F-V curve of the probes to more physiological range is marked in red. The fusion site corresponding to that of ArcLight-Q239 is highlighted with yellow. (TIF) [file pone.0081295.s001.tif]

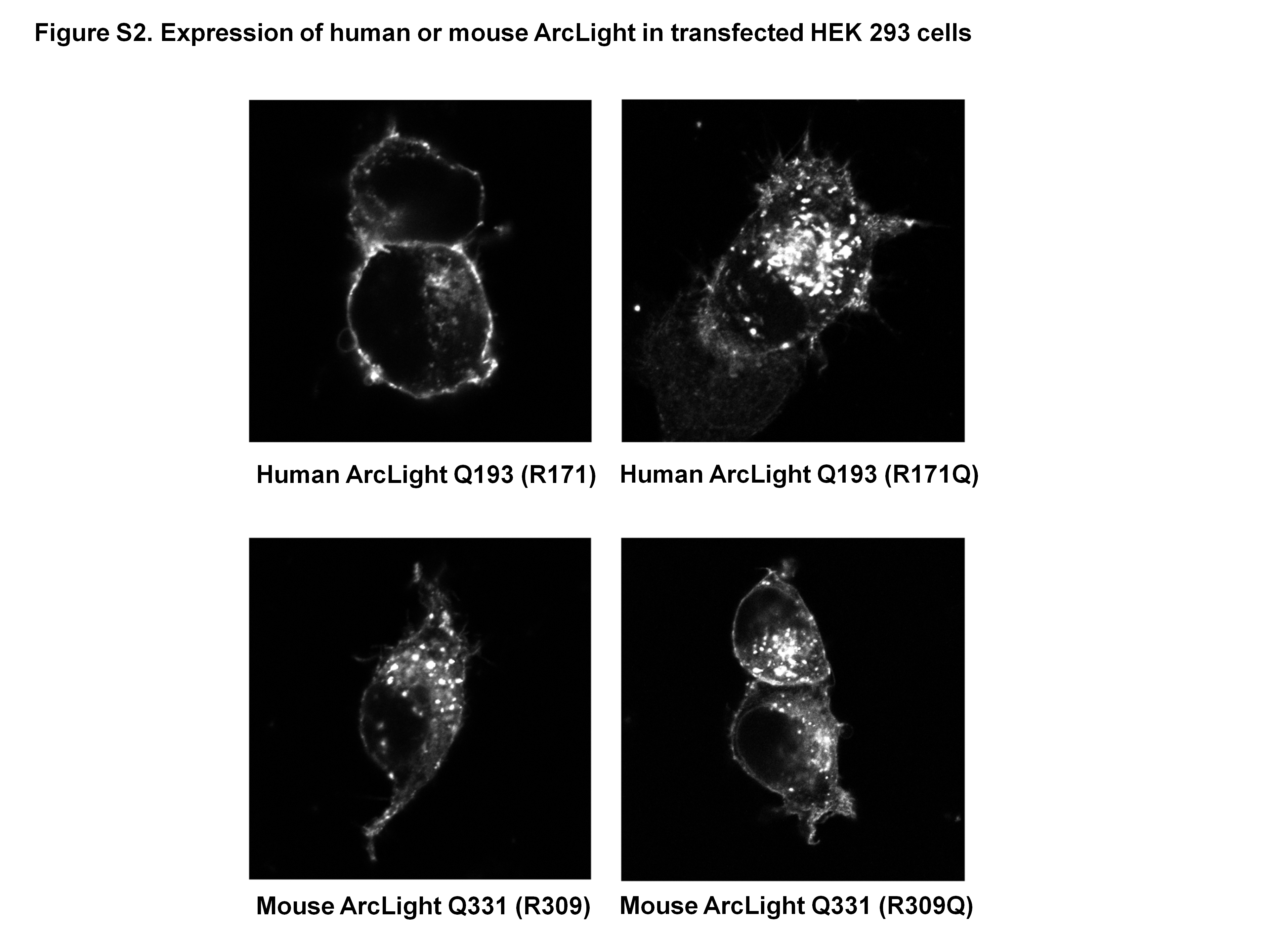

Supplement: Figure S2 — Expression of human or mouse ArcLight in transfected HEK 293 cells. Representative confocal images of human ArcLight-Q193 or mouse ArcLight Q331 expression in the HEK 293 cells. The probes carry either an arginine to glutamine mutation or not in the S4 domain. (TIF) [file pone.0081295.s002.tif]
